# Role of GABA pathway in motor and non-motor symptoms in Parkinson's disease: a bidirectional circuit

**DOI:** 10.1186/s40001-024-01779-7

**Published:** 2024-03-27

**Authors:** Bandar Alharbi, Hayder M. Al-kuraishy, Ali I. Al-Gareeb, Engy Elekhnawy, Hind Alharbi, Athanasios Alexiou, Marios Papadakis, Gaber El-Saber Batiha

**Affiliations:** 1 Department of Pharmacy Services, Prince Sultan Medical City (PSMMC), Riyadh, Saudi Arabia; 2 Department of Clinical Pharmacology and Medicine, College of Medicine, Mustansiriyah University, Baghdad, Iraq; 3 Pharmaceutical Microbiology Department, Faculty of Pharmacy, Tanta University, Tanta, Egypt; 4 Department of Pharmacy Services, Prince Sultan Medical City (PSMMC), Riyadh, Saudi Arabia; 5 Department of Science and Engineering, Novel Global Community Educational Foundation, Hebersham, NSW 2770 Australia; 6 AFNP Med, 1030 Vienna, Austria; 7 Department of Surgery II, University Hospital Witten-Herdecke, University of Witten-Herdecke, Heusnerstrasse 40, 42283 Wuppertal, Germany; 8 Department of Pharmacology and Therapeutics, Faculty of Veterinary Medicine, Damanhour University, Damanhour, AlBeheira Egypt; 9 University Centre for Research & Development, Chandigarh University, Chandigarh-Ludhiana Highway, Mohali, Punjab India; 10 Department of Research & Development, Athens, Funogen, 11741 Greece

**Keywords:** PD, GABA, Motor, Non-motor manifestations

## Abstract

Parkinson's disease (PD) is a progressive neurodegenerative disease as a result of the degeneration of dopaminergic neurons in the substantia nigra pars compacta (SNpc). The fundamental features of PD are motor and non-motor symptoms. PD symptoms develop due to the disruption of dopaminergic neurotransmitters and other neurotransmitters such as γ-aminobutyric acid (GABA). The potential role of GABA in PD neuropathology concerning the motor and non-motor symptoms of PD was not precisely discussed. Therefore, this review intended to illustrate the possible role of GABA in PD neuropathology regarding motor and non-motor symptoms. The GABA pathway is essential in regulating the inhibitory tone to prevent excessive stimulation of the cerebral cortex. Degeneration of dopaminergic neurons in PD is linked with reducing GABAergic neurotransmission. Decreasing GABA activity promotes mitochondrial dysfunction and oxidative stress, which are highly related to PD neuropathology. Hence, restoring GABA activity by GABA agonists may attenuate the progression of PD motor symptoms. Therefore, dysregulation of GABAergic neurons in the SNpc contributes to developing PD motor symptoms. Besides, PD non-motor symptoms are also related to the dysfunction of the GABAergic pathway, and amelioration of this pathway may reduce PD non-motor symptoms. In conclusion, the deregulation of the GABAergic pathway in PD might be intricate in developing motor and non-motor symptoms. Improving this pathway might be a novel, beneficial approach to control PD symptoms.

## Introduction

Parkinson's disease (PD) is a progressive neurodegenerative disease as a result of the degeneration of dopaminergic neurons in the substantia nigra pars compacta (SNpc) [1]. PD is the second worldwide neurodegenerative disease after Alzheimer's disease (AD). It affects 1–3% of the population worldwide > 65 years [2]. Notably, two types of PD are well-recognized: familial (genetic) PD and idiopathic (sporadic) PD; familial PD represents 10–15% of all PD types [3]. Many genes contribute to the pathogenesis of PD, including α-synuclein (*SNCN*), leucine-rich repeat kinase 2 (*LRRK2*), glucocerebrosidase (*GBA*), vacuolar protein sorting associated protein 35 (*VPS35*), phosphatase homolog-induced kinase (*PINK1*) and Parkinson protein 7 (*PAPK7*) [3, 4]. The interaction between the susceptible genes and environmental elements influences the onset of PD [5].

Different risk factors, such as sex, age, and ethnicity, contribute to the development of PD [9]. Old age is the leading factor that affects the onset severity of PD. Most PD cases have the age of 60–65 years; however, juvenile PD was reported at the age of less than 21 years [6]. PD is more prevalent in men and white occidental populations [6]. The black race has a lower PD incidence owing to the higher concentration of the neuroprotective neuromelanin [7].

**Fig. 1:**
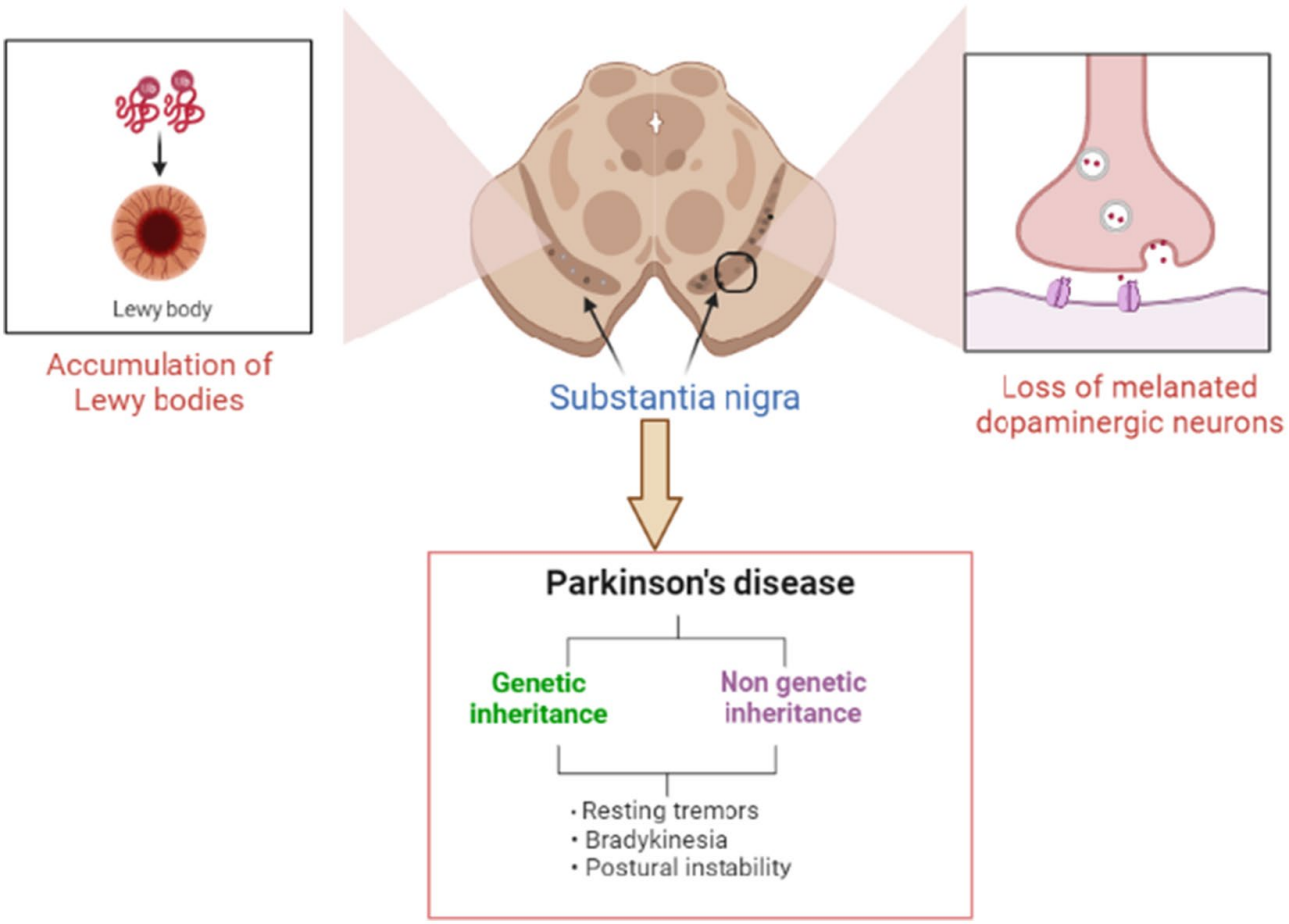
Pathophysiology of PD

Moreover, heavy metals such as iron, lead and manganese contribute to PD neuropathology via multiple mechanisms, including oxidative stress and mitochondrial dysfunction with subsequent synaptic dysfunction and disruption of brain neurotransmission [8]. Also, drug abuse such as cocaine increases the risk of PD through the induction of the dopaminergic neurons in the SNpc [9] (Fig. 1).

Neuronal cell death in the basal ganglion is the primary pathological event in PD. This is attributed to the deposition of α-synuclein [10]. Notably, normal α-synuclein is usually found in the presynaptic site and is significant in releasing neurotransmitters, including dopamine [11]. Genetic environmental factors trigger misfolding and aggregation of α-synuclein and the formation of Lewy bodies [12]. Such changes in neurons could provoke the death of neuronal cells and astrocytes with robust activation of microglia in the SNpc [13]. The accumulated α-synuclein activates degeneration of the dopaminergic neurons in the SNpc either directly due to the toxic impact of α-synuclein or indirectly through induction of mitochondrial dysfunction and proteasomal/lysosomal dysfunctions [14]. Remarkably, the neuropathology of PD occurs first in the olfactory bulb and medulla before affecting the SNpc [15]. The principal neuronal tracts connecting basal ganglia to the other brain regions are orbitofrontal, limbic, associative, oculomotor, and motor tracts are affected in PD neuropathology, causing both motor and non-motor symptoms [16]. Dopamine neurotransmitter released from the dopaminergic neurons is responsible for regulating motor activity. However, low dopamine level is connected with hypokinesia, while increasing dopamine activity leads to dyskinesia, highlighting a defect in motor activity due to dopamine activity and sensitivity [17].

The primary clinical characteristics of PD are motor symptoms such as bradykinesia, postural instability, rigidity, and resting tremors that start when more than 70% of the dopaminergic neurons in the SNpc are damaged [18]. Non-motor symptoms, such as autonomic dysfunction, anosmia, constipation, sleep disorders, and cognitive dysfunction, are usually initiated before the motor symptoms by decades [19]. PD symptoms are correlated to the disturbance of various neurotransmitters, such as dopamine, acetylcholine (Ach), and γ-aminobutyric acid (GABA) [20]. There is little information regarding the role of GABA in PD neuropathology, mainly concerning the development of motor and non-motor symptoms. Thus, this review aimed to clarify the potential role of GABA in PD regarding motor and non-motor symptoms.

### GABA overview

**Fig. 2:**
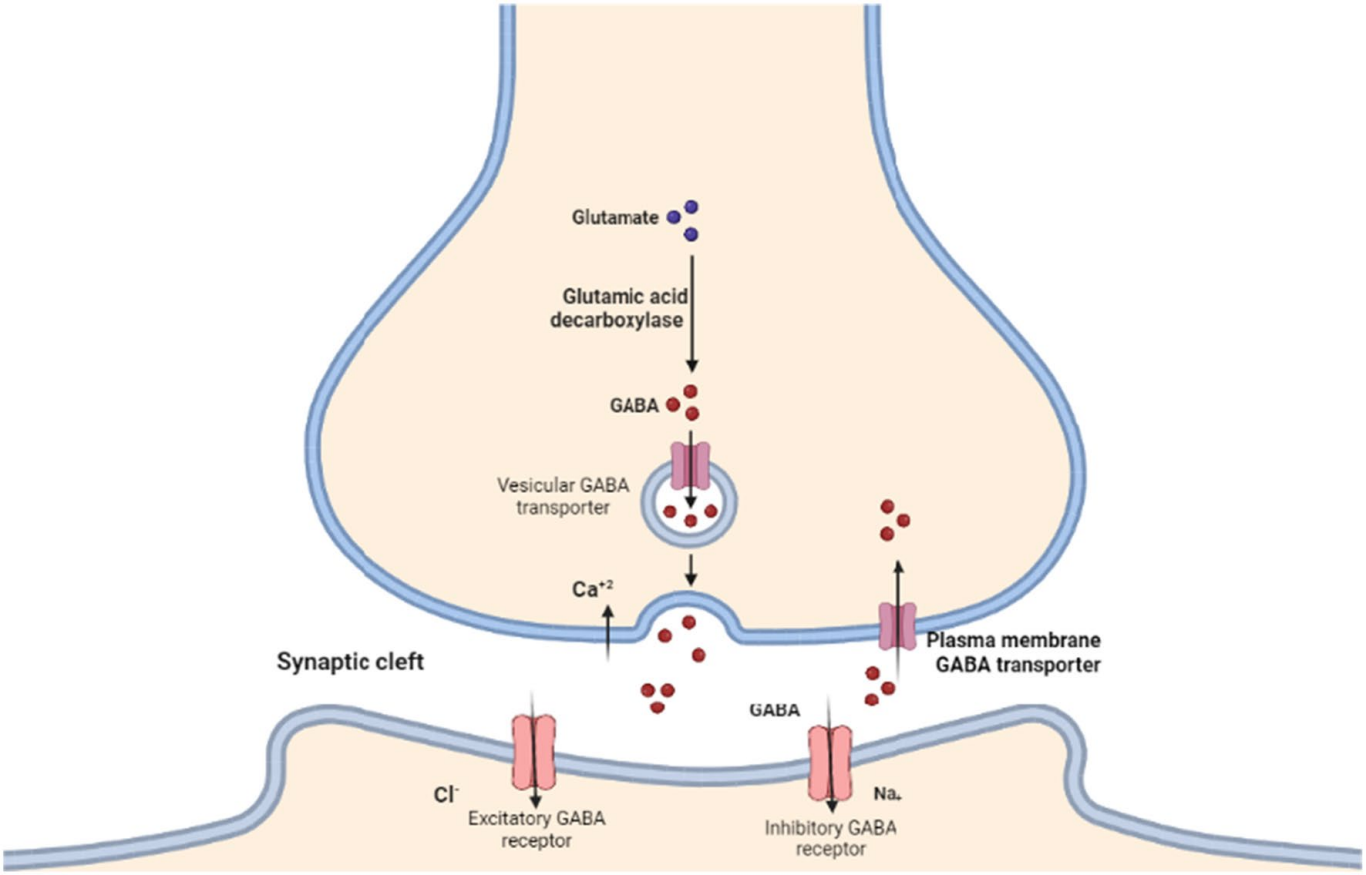
GABA and GABA receptors: glutamate is converted to GABA by the enzyme glutamic acid decarboxylase, which is then stored in the specialized vesicles. The GABA is released upon presynaptic activation of GABAergic neurons, which activates Cl- ion entrance through activation of GABA receptors, leading to hyperpolarization and inhibition of the postsynaptic membrane. Excess GABA in the synaptic cleft is reuptake through GABA transporter to the presynaptic neurons

GABA is a multi-functional molecule in the CNS, peripheral nervous system (PNS) and non-neuronal tissues [21]. GABA is an inhibitory neurotransmitter extensively expressed in the central nervous system (CNS) [22]. GABA acts on the GABA receptors comprising GABA_A_, GABA_B_ and GABA_C_ [23]. GABA_B_ is a G-protein metabotropic receptor, while GABA_A_ and GABA_C_ are Cl-gated channels [24] (Fig. 2).

GABA receptor comprises three central subunits: α, β and γ in a heteromeric or homomeric fashion [22]. GABA regulates neuronal activity through the opening of K^+^ or inhibiting of Ca^+2^ via the G-protein signaling pathway [23]. GABA is synthesized from glutamate by the enzyme glutamic acid decarboxylase (GAD); the formed GABA is transported to the presynaptic terminals and stored in specialized vesicles [23]. GAD is widely expressed in the CNS inhibitory neurons and associated with GABA neurons. Dysregulation of GAD is linked with the development of schizophrenia and epilepsy [25].

Depolarization of presynaptic GABergic neurons activates the release of GABA into synaptic space with succeeding stimulation of post-GABA receptors and postsynaptic inhibition [23]. In addition, GABA from the synaptic cleft may leak outside and activate extra-synaptic GABA receptors, causing tonic inhibition [25]. Mainly, GABA_A_ in the dorsal horn is found in presynaptic and postsynaptic neurons, mediating presynaptic inhibition and primary afferent depolarization correspondingly [26]. Extra-synaptic α5GABA_A_ on the proprioceptive afferent neurons leads to tonic depolarization of the spinal cord via modulation of Na^+2^ channels [26]. Meaningfully, GABAergic neurons are excitatory during prenatal and postnatal periods; however, these neurons undergo developmental changes from excitatory to inhibitory [27]. *K* + -Cl mainly mediates the polarity of GABA neurons to inhibitory functions^−^ co-transporter (KCC2) [27]. Oxytocin plays a critical role in the transition of GABAergic neurons to inhibitory processes through modulation of KCC2 [28]. In the absence of oxytocin, the activity of KCC2 is reduced with an increase in hyper-excitability state and related disorders like epilepsy and neurodevelopmental disorders [28]. Besides, expressions of GABA_A_ receptors are regulated by allopregnanolone steroid, which exerts positive and negative impacts in acute and chronic effect correspondingly on the expression of GABA_A_ receptors and the progression of dementia [29].

Furthermore, dysfunction of GABAergic neurons in the frontal lobe increases the risk of developing frontotemporal dementia [30]. Restoration of GABAergic neurotransmission by GABA transporter inhibitor tiagabine prevents the development of frontotemporal dementia in both animals and humans [30]. These findings indicated the potential role of GABAergic neurons in regulating neuronal inhibition and preventing neuronal hyper-excitability.

### GABA pathway and neurodegenerative disorders

GABAergic neurons are intricate in regulating memory and learning, which are significant variables of AD [31]. Progressive neuronal accumulation of Aβ distorts synaptic function and induces the progression of cognitive impairment through dysregulation of GABAergic neurotransmission [31]. Interaction of Aβ with the neurotransmission network in brain areas linked to memory, such as the hippocampus and amygdaloidal complex, leads to memory impairment and cognitive dysfunction [32]. Regulation of cognitive function is mostly fine-tuning between the excitatory neurotransmitters and GABA inhibitory systems [33]. Hippocampal and cortical functions mainly depend on the GABA inhibitory function to maintain the activity of synaptic plasticity [34]. Hippocampus GABA inhibitory neurons represent 10–15% of total brain inhibitory neurons [34]. It has been revealed that GABAergic neurons are significantly dysregulated and intricate in the pathogenesis of AD [32, 33]. In AD, excitatory neurotransmitters are involved in the pathogenesis of Aβ and tau deposition due to reduced protective GABA inhibitory function [31]. Consequently, disruption of the inhibitory/excitatory axis in the brain could be a possible mechanism for the progress of seizures in AD patients [35].

Indeed, GABAergic neurons are highly distressed in various neurodegenerative diseases, like AD and could be a therapeutic goal in controlling such disorders [31]. A study explained that the density of GABAergic neurons declined next to the Aβ plaques [31], proposing the toxic impacts of Aβ plaques on GABAergic neuron viability. In the AD mice model, it was found that the Aβ plaques are much more harmful to the hippocampal GABAergic neurons compared to other neurons [36]. An in vitro demonstrated that Aβ has differential neurotoxicity for GABAergic neurons [37]. Aβ triggers cell membrane perforation with increasing Ca + 2 effluxes in the hippocampal GABAergic neurons with disturbances of excitatory–inhibitory synaptic function [37]. In AD patients, the concentration of GABA is highly abridged in the temporal cortex and other brain regions [38]. Furthermore, the level of GABA is declined in the cerebrospinal fluid (CSF) in AD [38].

Moreover, deficiency of GABA is associated with the development of Huntington's chorea and other neurodegenerative disorders, as well as neuropsychiatric disorders like panic, depression, and anxiety [39]. Likewise, inflammatory reactions in multiple sclerosis (MS), mainly IL-1β, inhibit GABA function with significant alterations of the GABA pathway [40]. A study observed that CSF GABA declined in MS patients concerning controls [41]. Alteration of GABAergic neurons and reduction of GABA levels are also related to the severity of cognitive deficiency in MS patients [41]. Furthermore, the GABA pathway is highly deregulated in amyotrophic lateral sclerosis (ALS), leading to cortical hyper-excitability [42]. A cross-sectional study revealed reduced GABAergic neurons in ALS patients, causing more severe motor alterations [42].

These findings proposed that the GABA pathway is highly dysregulated in patients with neurodegenerative disorders, and targeting this pathway could be a potential therapeutic strategy against the development and progression of neurodegenerative disorders.

### Role of GABA pathway in PD

GABA pathway plays a critical role in regulating inhibitory tone on the globus pallidus (GP), SNpc, and thalamus, preventing excessive stimulation of the cerebral cortex [43]. Dysregulation of the GABA pathway in PD triggers neuronal hyper-excitability, leading to dyskinesia or bradykinesia [43, 44]. Deregulation of the GABA pathway may be involved in developing and progressing motor and non-motor manifestations in PD [44].

### GABA and motor manifestations of PD

The mechanism of motor dysfunction in PD is well-defined due to dopaminergic dysfunction; however, alteration of other neurotransmitters like serotonin, Ach, glutamate, noradrenaline, and GABA are also involved in PD neuropathology [45, 46]. GABAergic signaling controls cognition, information processing, and sensory perception [46]. Cardinal motor dysfunction in PD, like resting tremor, bradykinesia, and rigidity, are developed due to the degeneration of dopaminergic neurons in the SNpc. In advanced PD, dyskinesia and motor fluctuation progress due to the degeneration of non-dopaminergic pathways [46].

Disturbance of GABAergic neurons had been reported in the basal ganglia at the postmortem of PD [47]. Tritsch et al. [48] confirmed that dopamine is co-released with GABA from dopaminergic neurons independent of vesicular GABA transporters. The release of GABA also requires the activation of vesicular monoamine transporter 2 (VMAT2), which is also a neurotransmitter of dopamine [48]. Interestingly, the expression of VMAT2 on the GABAergic neurons has an essential role in the release of GABA [49]. Besides, dopaminergic neurons in the SNpc inhibit the striatum via presynaptic activation of GABA receptors [49]. The dopaminergic neurons obtain GABA through presynaptic uptake and then release with dopamine by GABA transporters [49]. Increasing striatal input due to a deficiency of GABA leads to the development of bradykinesia in PD [50]. Selective depletion of dopaminergic receptor 2 (D2R) from an indirect pathway leads to severe motor impairment in mice by decreasing GABAergic neurotransmission [50]. Therefore, D2R is essential for GABAergic neurotransmission and motor control. These observations suggest a mutual interaction between dopamine and GABA. Thus, degeneration of dopaminergic neurons is linked with reducing GABA levels in PD.

It has been reported that the GABA pathway is highly dysregulated in PD neuropathology by disturbing Ca^2+^ cellular signaling. GABA/Ca^2+^ maintains neuronal activity in the CNS by preventing intracellular deposits of proteins, Ca^2+^ and Lewy bodies [51]. Excessive Ca^2+^ accumulations stimulate α-synuclein aggregation and injury of dopaminergic neurons through induction of excitotoxicity and apoptosis, leading to the development of PD [52]. Notably, the diminution of inhibitory GABAergic neurons triggers the over-activation of cholinergic neuronal signaling, leading to progressive muscle contraction of both agonist and antagonist muscles with the development of stiffness and postural instability [53]. The hyperpolarization of GABAergic neurons regulates presynaptic neurotransmission and prevents neuronal hyper-excitability by maintaining Ca^2+^ homeostasis [53]. This effect attenuates Ca^2+^ dyshomeostasis-induced dopaminergic neuron injury. Dopaminergic neurons are highly susceptible to the neurotoxic effects of α-synuclein due to higher expression of Ca^2+^ voltage-gated channels [54]. Ca^2+^ voltage-gated channels improve the release of α-synuclein in vitro and in vivo with subsequent aggregation and development of synucleinopathies [54]. Therefore, regulation of Ca^2+^ voltage-gated channels by GABA may prevent Ca^2+^-induced excitotoxicity, oxidative stress, mitochondrial dysfunction and development of PD. Reduction of GABA promotes oxidative stress and mitochondrial dysfunction, which is linked with PD neuropathology [55]. Of note, neurosteroids which promote the synthesis of neuronal GABA are highly reduced in PD and other neurodegenerative ailments, leading to excitotoxicity and apoptosis [64]. Experimental studies showed that dysregulation of GABAergic neurons in the SNpc leads to abnormal neuronal firing in rat PD models [65]. A recent study demonstrated that induction of GABAergic neuron generation by astrocyte reprogramming improves motor symptoms in experimental PD [66]. A recent study revealed that presynaptic inhibition in the motor cortex is highly reduced in PD patients, which may explain PD's non-dopaminergic feature [67]. A study exhibited that GABAergic neuron activity in the upper brainstem is reduced compared to the controls [68]. Thus, restoring GABA activity by GABA agonists can attenuate motor symptoms in the PD model [56]. GABA agonists like baclofen and acamprosate protect dopaminergic neurons and striatal terminals from oxidative stress in 6-hydroxydopamine (6-OHDA)-induced PD in rats [56]. Combining baclofen and acamprosate inhibits glutamatergic neurotransmission, improving GABAergic neurotransmission and suppressing neuronal apoptosis and neuroinflammation [56]. A preclinical study observed that GABA_B_ receptor agonist baclofen attenuates motor deficits in MPTP-induced PD in rats by improving GABAergic neurotransmission in the SNpc. Supporting this finding, GABA_B_ receptor antagonist CGP35348 reverses the protective effect of baclofen in MPTP-induced PD in rats [58]. Likewise, Lozovaya et al. [57] showed that the inhibitory effects of GABAergic neurons regulate cholinergic excitatory drive. Consequently, improving inhibitory GABAergic neurons by GABA agonists may reduce the severity of motor symptoms in PD [57]. Bumetanide is an antagonist of chloride importer that improves brain inhibition by reducing intracellular chloride and increases GABAergic neurotransmission in PD patients [59]. Therefore, the augmentation activity of GABAergic neurons may improve motor symptoms in PD. GABA_A_ agonist zolpidem has a remarkably beneficial effect in reducing dyskinesia even after a single dose in PD patients [60]. Zolpidem has a peculiar effect on movement disorders in PD patients, as the use of other GABA_A_ receptor agonist hypnotics like zopiclone and triazolam produced no beneficial motor effects in women with PD [60]. Bohen et al. [61] found that a reduction of GABAA receptor expression in the thalamus correlates with motor dysfunction in PD patients [61]. Conversely, tremor, rigidity, and bradykinesia are developed due to GABAergic neuron hyperactivity driven by GP on the thalamus, and thalamocortical GABAergic neuron activity is increased in PD, as documented in a case–control study [62]. Motor cortex GABA level is inversely correlated to PD disease so that GABA depletion may participate in the development of motor symptoms [62]. However, a case–control study observed that GABA concentration was greater in pons concerning the putamen in the early PD [63], suggesting that altering GABAergic inhibitory tone in the brainstem could be an early neuropathological finding in PD. Therefore, administration of the GABAA receptor antagonist flumazenil has improved postural instability in PD patients [69]. In addition, the GABA_A_ receptor antagonist restores dopaminergic firing and regulates motor dysfunction in PD mouse model [70]. Interestingly, dysregulation of GABA_A_ receptor expression is differentially affected in PD increased in the cerebral cortex and reduced in other brain regions [71], signifying a specific alteration of GABA_A_ receptor expression rather than generalized dysregulation.

These findings suggest that dysregulation of GABAergic neurons in the SNpc contributes to the development of motor symptoms in PD, and targeting this pathway could be a novel approach to managing the motor symptoms of PD.

### GABA and non-motor manifestations of PD

Non-motor manifestations in PD, like cognitive dysfunction, sleep disorders, olfactory dysfunction, gastrointestinal disorders, and visual disturbances, represent the primary source of PD burden [72]. These manifestations usually occur many years before the development and progression of motor symptoms. The underlying mechanism for developing these manifestations could be related to GABAergic dysfunction [73]. It has been shown that disturbance of GABAergic neurons contributes to the development of non-motor symptoms in early PD as GABA is co-released with dopamine in the striatum. Thus, the degeneration of the dopaminergic neurons in the SNpc affects GABAergic neurotransmission [74]. Firbank and his colleagues [75] found that GABA concentration was reduced in the occipital cortex, leading to visual hallucination. A cohort study involving 39 PD patients, 19 with hallucination and 17 without hallucination, showed that GABA concentration measured by magnetic resonance spectroscopy was reduced in the occipital cortex and correlated with excitability in PD patients with hallucination [75]. It has been shown that PD patients had visual disturbances with abnormal color vision in the late stage due to alteration of retinal GABAergic neurons [76]. Depletion of retinal GABAergic neurons leads to the development of visual disturbances [77]. Remarkably, increasing retinal GABAergic neurons by GABA agonists also induces visual disturbances [78]. Thus, an optimal GABA level is essential for accuracy and discrimination. GABAergic neurons regulate visual perception; deregulation of GABAergic neurons is engaged with developing visual disturbance in PD [79]. Visual disturbances and retinal abnormalities are observed in PD patients and animals due to the deposition of α-synuclein in the retina [80]. The development of visual hallucination in PD is complex and may be related to anti-PD medications such as anticholinergic benzhexol [111]. Therefore, the management of visual hallucination in PD is not merely associated with the dysregulation of brain GABA since it is related to other neurotransmitters [111].

Indeed, olfactory disorders are common in PD, and more than 90% of PD patients have this disorder [81]. A study illustrated that the olfactory bulb volume was smaller than the matched controls [81]. The reduced volume of the olfactory bulb is correlated with the reduced volume of the putamen and hippocampus [81]. A postmortem study revealed the volume of olfactory bulbs is reduced in PD patients compared to healthy controls [82]. Notably, microstructural changes in the olfactory bulb correlate with dopaminergic neuron dysfunction in the putamen [82]. Olfactory dysfunction in PD is correlated with neuronal loss and structural changes in the nucleus basalis, raphe nuclei, and locus coeruleus [83]. These neuroanatomical changes suggest the involvement of serotonergic, noradrenergic, and cholinergic in olfactory dysfunction [83]. GABAergic neurons in the olfactory pathway regulate odor perception and sensitivity [83]. The development of aberrant GABAergic neurons is associated with olfactory dysfunction in the AD mice model [84]. These findings proposed that dysfunction of GABAergic neurons in PD could be the primary mechanism for developing olfactory dysfunction. In this state, the potentiation of GABAergic neurons may mitigate olfactory dysfunction in PD. A recent experimental study demonstrated that GABA agonist muscimol improves olfactory dysfunction in mouse AD model by regulating presynaptic GABA release and improving of GABAergic neurotransmission in the olfactory system [85]. As well, the development of olfactory dysfunction in PD increases the risk of the development of dementia [86]. GABAergic neurotransmission in the olfactory system modulates glutamatergic neurotransmission in the prefrontal cortex, which is implicated in the pathogenesis of PD [85, 86]. Therefore, early recognition and management of olfactory dysfunction may prevent PD-related complications like dementia. It has been shown that benzodiazepine receptors improve the functional activity of olfactory neurons, and activation of these receptors by GABA_A_ receptor agonists can improve olfactory dysfunction [84]. Activation of the olfactory via low oscillation pulse reduces Aβ accumulation and cognitive impairment in mice by increasing the expression of GABA_A_ receptors [87], suggesting that dysfunction of GABAergic neurotransmission in the olfactory system is implicated in the development of cognitive impairment. However, aberrant expression of the GABA_A_ receptor is involved in the development of cognitive dysfunction in PD mouse model by increasing the accumulation of α-synuclein in early PD. In addition, α-synuclein inhibits the release of GABA in the interneurons, leading to olfactory and cognitive dysfunction in PD mouse model [88].

Furthermore, cognitive dysfunction is frequently associated with PD in about 20–25% [87]. It has been reported that PD patients had a greater risk for the development of dementia and cognitive dysfunction compared to the controls [87]. PD-induced cognitive dysfunction and dementia are developed due to cholinergic deficit, α-synuclein-induced neuronal injury, and dysmetabolism [87, 88]. Single nucleotide polymorphisms (SNPs) RYR2SNP rs10495397 in the Korean population is the most frequent SNP-linked development of cognitive impairment with PD through the development of neuroinflammation [88]. Cognitive dysfunction in PD may develop due to dysregulation of various neurotransmitters like Ach and dopamine in the frontostriatal pathway [89]. Cognitive dysfunction in PD is correlated with both motor and non-motor symptoms [90]. Of note, somatostatin-expressing GABAergic neurons have excitatory effects on the cortical circuits regulating neuronal activity [91].

Therefore, dysfunction of GABAergic neurons is associated with the development of cognitive dysfunction. It has been reported that GAD expression was reduced in PD patients, reducing the neuronal synthesis and release of GABA [92]. Findings from a postmortem study involving 19 PD patients and 19 healthy controls showed that GAD67 expression was decreased in the prefrontal cortex of PD patients compared to controls [92]. Nutt et al. [93] observed that increased expression of the *L-amino acid decarboxylase* gene by VY-AADC01 improves response to L-dopa therapy in PD patients by enhancing GABA activity. In addition, GABA activity is reduced in PD patients during cognitive stress and stimulation [86].

Furthermore, blunted GABA response to dopamine agonists in PD patients leads to behavioral and cognitive abnormalities [86]. These observations suggest that GABAergic dysfunction in PD is linked with the progression of cognitive dysfunction. Thus, augmentation of GABAergic activity by GABA_A_ agonists could be effective in mitigating cognitive dysfunction in PD. However, the antiepileptic GABA transaminase inhibitor vigabatrin did not improve the cognitive function in epileptic patients [94]. Also, tiagabine, which inhibits GABA transporter, can protect dopaminergic neurons in the SNpc and enhance cognitive function in mouse PD models by inhibiting microglial activation [95]. Moreover, GABA transaminase inhibitor valproate, which is commonly used in the management of epilepsy, its long-term use is associated with the development of cognitive impairment.

Conversely, a recent experimental study found valproate has a neuroprotective role and enhances cognitive function in mice with experimental stroke by increasing the release of GABA and enhancing long-term potentiation. It has been shown that valproate improves cognitive function and attenuates degeneration of the dopaminergic neurons in the SNpc by 50% in rotenone-induced PD through inhibition of histone deacetylase, increasing the accumulation of α-synuclein [96]. However, prolonged use of valproate increases PD risk by inducing progressive degeneration of the dopaminergic neurons in the SNpc by alternating the expression of genes involved in PD neuropathology. A recent clinical trial illustrated that most antiepileptic drugs increase PD risk [97]. Thus, GABA-enhancing drugs have conflicting outcomes on cognitive function and PD risk.

Furthermore, PD neuropathology is associated with sleep disorders, which were reported to be up to 98% in PD patients. Sleep disorders like insomnia, daytime sleepiness, sleep fragmentation, restless leg syndrome and REM behavior disorder (RBD) are frequently developed in the early stage of PD [94]. In addition, sleep disorders adversely affect cognitive function in PD patients [95]. A meta-analysis and systematic review showed that sleep disorders, mainly RBD, are associated with cognitive dysfunction [95]. Remarkably, sleep disorders increase the severity and progression of PD by enhancing the release and deposition, and reducing the clearance of α-synuclein [96]. Sleep disorders in PD are due to the reduced activity of GABAergic neurons [97]. Therefore, activation of the GABAergic pathway by benzodiazepines like nitrazepam could be effective in the management of sleep disorders in PD [98]. A clinical trial indicated that benzodiazepine clonazepam is well-tolerated in improving sleep disorders in PD patients [99]. A scoping review illustrated that clonazepam effectively restored normal sleep in PD patients by enhancing brain GABAergic neurotransmission. Zolpidem is effective for insomnia in PD patients through modulation of the GABAergic pathway; it decreases latency for NREM sleep [99]. It has been reported that zolpidem was very effective in treating insomnia in PD patients owing to its short half-life without daytime sleepiness [100]. A systematic review showed that zolpidem, through potentiation of comprised GABergic neurotransmission, is also effective for PD and other movement disorders. Similarly, a clinical trial showed that daily treatment with benzodiazepine receptor agonist eszopiclone improves sleep quality in PD patients by potentiating brain GABA effects [101]. Therefore, these findings proposed that dysregulation of the GABAergic pathway plays a crucial role in the development of sleep disorders in PD, and activation of the GABA pathway may regulate sleep patterns and attenuate the development of sleep disorders.

Furthermore, dysregulation of the GABAergic pathway is linked with the development of neuropsychiatric disorders like depression and anxiety [100]. Notably, somatostatin-expressing GABAergic neurons are reduced in PD patients with *Parkin* gene mutation [101]. In the CNS, somatostatin is highly co-localized with GABAergic neurons; it acts as a neuromodulator or co-neurotransmitter and regulates the functional activity of these neurons. CSF somatostatin level reflects the density and activity of GABAergic neurons [102]. Different studies have shown that the CSF somatostatin level was reduced in PD [102, 103]. A recent systematic review and meta-analysis showed that PD neuropathology is associated with functional and structural changes in the neuronal circuits involved in the pathogenesis of anxiety and motor deficits [104]. Likewise, a systematic review and meta-analysis illustrated that depression is found in 38% of PD patients and was more associated with the female sex and *GBA1* gene mutation [105]. Also, α-synuclein depression is regarded as independent non-motor symptoms in PD that appear in the early stage and continue throughout the disease duration [105]. Luscher et al. [106] hypothesized that dysfunction of the GABAergic pathway was linked with the development of depressive disorders. A study observed that CSF GABA was low in depressed patients compared to the controls [107]. A systematic review revealed that CSF levels of GABA, somatostatin and brain-derived neurotrophic factor (BDNF) are reduced in patients with depression. Likewise, the reduction of plasma GABA is associated with the development of anxiety disorders [108]. These findings suggest that dysfunction of the GABAergic pathway is related to the development of depression and anxiety in PD. Hence, improvement of brain GABA neurotransmission could be an effective therapeutic strategy in treating neuropsychiatric disorders such as depression and anxiety in PD. It has been stated that the antiepileptic drug levetiracetam enhances neuronal GABA release. A clinical trial disclosed that levetiracetam improves anxiety disorders by enhancing GABA neurotransmission [109]. A double-controlled clinical trial demonstrated that levetiracetam improves cognitive impairment, neuropsychiatric disorders and motor deficits in PD patients. In addition, tiagabine attenuates the development of neuropsychiatric manifestations in experimental rats through the modulation of GABA neurotransmission [110]. These verdicts indicated that deregulation of GABAergic neurotransmission in PD is implicated in the development of neuropsychiatric manifestations such as depression and anxiety. Thus, enhancement of GABAergic neurotransmission by GABA agonists and GABA modulators could be a therapeutic strategy in the management of neuropsychiatric disorders in PD.

Finally, gastrointestinal (GIT) disturbances, including constipation, gastroparesis, nausea, vomiting and hypersalivation, are common in PD due to dysfunction of the enteric nervous system (ENS) and degeneration of the vagus nucleus in the brainstem [108]. The GABAergic pathway regulates intestinal motility and peristaltic reflex [109]. All types of GABA receptors are highly expressed in the GIT and regulate excitatory and inhibitory signaling in the ENS, neuroimmune interaction, and GIT inflammation [109]. GABA receptor agonists can improve GIT disturbances and inflammation in mice [109]. Notoriously, GABA at low concentration exerts an inhibitory effect, while higher concentration leads to an inhibitory effect on the GIT peristaltic activity [110]. GABA_A_ agonist muscimol excites the GIT peristaltic activity blocked by GABA_A_ antagonist bicuculline [110]. Therefore, GABA is regarded as a modulator of colonic peristalsis through modulation of Ach release from enteric neurons [110]. Librium is a well-known benzodiazepine used in the management of gastrointestinal disorders through the activation of GABA signaling in the GIT [111].

These observations indicated that dysfunction of the GABAergic pathway in PD is implicated in the GIT disturbances. Thus, non-motor manifestations in PD are developed due to dysfunction of the GABAergic pathway and amelioration of this pathway may reduce PD severity related to non-motor symptoms.

Also, the miRNAs present at the synapse play a crucial role in the regulation of local synaptic proteins and synapse function. Several miRNAs have been identified to regulate the key proteins of the GABA system in various neurodegenerative diseases. A previous research [112] has revealed a molecular connection between the regulation of GABAergic synapse function by synapse miRNA in AD. However, information on this important aspect needs more research. Taken together, targeting synapse miRNAs to modulate the GABA function could be a novel approach to restoring synapse function in AD and other neuropsychiatric disorders. However, further research is still required to fully understand the association between synaptic miRNAs and GABAergic synapse function.

Taken together, dysregulation of the GABAergic pathway in PD could be involved in the development and progression of motor and non-motor symptoms in PD, and enhancement of this pathway by GABA agonists could be an effective therapeutic modality in the management of PD (Tables 1, 2).

**Table 1:** Human studies revealing the potential role of GABA and effects of GABA modulators on the motor and non-motor symptoms of PD

A study type	Findings	Refernces
Postmortem study	Disturbance of GABAergic neurons in the basal ganglia in PD patients	[47]
A case series	GABA_B_ receptor agonist baclofen attenuates motor deficits in MPTP-induced PD in rats	[58]
A case–control	Bumetanide increases GABAergic neurotransmission in PD patients	[59] [60]
A case–control	Zolpidem reduces dyskinesia in PD patients	[62]
A case–control	Thalamocortical GABAergic neuron activity is increased in PD patients	[63]
A case–control	GABA concentration was more significant concerning the GABAA receptor antagonist flumazenil, which improves postural instability in PD patients	[85]
A cohort study		[113]
A case–control	Aβ accumulation and cognitive impairment in mice by increasing the expression of GABA_A_ receptors	[114]
A case–control	GAD67 expression was decreased in the prefrontal cortex of PD patients compared to controls	[115]
A clinical trial	Valproate improves cognitive function and attenuates degeneration of the dopaminergic neurons in the SNpc	[116–118]
A clinical trial	Clonazepam restores normal sleep in PD patients	[119, 120]
A clinical trial	Zolpidem is effective for insomnia in PD patients	[121, 122]
A clinical trial	Eszopiclone improves sleep quality in PD patients	[99]
A clinical trial	Levetiracetam improves neuropsychiatric disorders and motor deficit in PD patients	[123–129]

**Table 2:** Animal studies revealing the potential role of GABA and effects of GABA modulators on the motor and non-motor symptoms of PD

A study type	Findings	Refs.
Rats	Dysregulation of GABAergic neurons in the SNpc leads to abnormal neuronal firing in rat PD models	[65]
Rats	GABA agonists protect dopaminergic neurons and striatal terminals from oxidative stress in 6-OHDA-induced PD in	[56]
Rats	Most antiepileptic drugs increase PD risk	[130]

## Conclusions

PD is characterized by motor and non-motor symptoms developed in response to the disruption of neurotransmitters, including GABA. The GABA pathway is extremely deranged in PD patients. The GABA pathway plays a role in regulating inhibitory tone to prevent excessive stimulation of the cerebral cortex. Degeneration of dopaminergic neurons is associated with reducing GABAergic neurotransmission in PD. Reduction of GABA promotes oxidative stress and mitochondrial dysfunction associated with PD neuropathology. Therefore, restoring GABA activity by GABA agonists can attenuate PD motor symptoms. Hence, dysregulation of GABAergic neurons in the SNpc contributes to developing PD motor symptoms. Also, non-motor symptoms in PD are generated due to dysfunction of the GABAergic pathway and amelioration of this pathway may reduce PD severity related to non-motor symptoms.

The deregulation of the GABAergic pathway in PD might be intricate in developing motor and non-motor symptoms of PD. Enhancing this pathway by GABA agonists could be a new therapeutic modality in managing PD. Targeting the GABA pathway might be a novel therapeutic strategy in managing motor and non-motor manifestations in PD.

## Data Availability

All data are available in the manuscript.
